# Evaluation of the TCR Repertoire as a Predictive and Prognostic Biomarker in Cancer: Diversity or Clonality?

**DOI:** 10.3390/cancers14071771

**Published:** 2022-03-31

**Authors:** Andrea Aran, Laia Garrigós, Giuseppe Curigliano, Javier Cortés, Mercè Martí

**Affiliations:** 1 Immunology Unit, Department of Cell Biology, Physiology and Immunology, Institut de Biotecnologia I Biomedicina (IBB), Universitat Autònoma de Barcelona (UAB), 08193 Bellaterra, Spain; andrea.aran@uab.cat; 2 International Breast Cancer Center (IBCC), 08017 Barcelona, Spain; laia.garrigos@ibcc.clinic (L.G.); jacortes@vhio.net (J.C.); 3 Division of Early Drug Development, European Institute of Oncology, IRCCS, 20141 Milano, Italy; giuseppe.curigliano@ieo.it; 4 Department of Oncology and Hemato-Oncology, University of Milano, 20122 Milano, Italy; 5 Medica Scientia Innovation Research (MedSIR), 08018 Barcelona, Spain; 6 Medica Scientia Innovation Research (MedSIR), Ridgewood, NJ 07450, USA; 7 Department of Medicine, Faculty of Biomedical and Health Sciences, Universidad Europea de Madrid, 28670 Madrid, Spain

**Keywords:** clonality, diversity, immune-checkpoint inhibitor, neoantigens, TCR, TILs, tumour-associated antigens

## Abstract

**Simple Summary:**

The TCR is the T cell antigen receptor, and it is responsible of the T cell activation, through the HLA-antigen complex recognition. Studying the TCR repertoire in patients with cancer can help to better understand the anti-tumoural responses and it has been suggested to have predictive and or/prognostic values, both for the disease and in response to treatments. The aim of this review is to summarize TCR repertoire studies performed in patients with cancer found in the literature, thoroughly analyse the different factors that can be involved in shaping the TCR repertoire, and draw the current conclusions in this field, especially focusing on whether the TCR diversity—or its opposite, the clonality—can be used as predictors or prognostic biomarkers of the disease.

**Abstract:**

T cells play a vital role in the anti-tumoural response, and the presence of tumour-infiltrating lymphocytes has shown to be directly correlated with a good prognosis in several cancer types. Nevertheless, some patients presenting tumour-infiltrating lymphocytes do not have favourable outcomes. The TCR determines the specificities of T cells, so the analysis of the TCR repertoire has been recently considered to be a potential biomarker for patients’ progression and response to therapies with immune checkpoint inhibitors. The TCR repertoire is one of the multiple elements comprising the immune system and is conditioned by several factors, including tissue type, tumour mutational burden, and patients’ immunogenetics. Its study is crucial to understanding the anti-tumoural response, how to beneficially modulate the immune response with current or new treatments, and how to better predict the prognosis. Here, we present a critical review including essential studies on TCR repertoire conducted in patients with cancer with the aim to draw the current conclusions and try to elucidate whether it is better to encounter higher clonality with few TCRs at higher frequencies, or higher diversity with many different TCRs at lower frequencies.

## 1. Introduction

The role of the immune system in cancer has gained considerable interest in the last few years, especially after the development of first immunotherapies. Currently, increasingly more treatments are based on immunotherapy, such as the immune checkpoint inhibitors (ICI), the adoptive cell transfer, or the CAR-T cell treatments. Moreover, different components of the immune system and their changes or evolutions can be used as biomarkers or may have a prognostic value for the disease. In particular, the study of the presence of tumour-infiltrating lymphocytes (TIL) is now widely used in patients with cancer since a high level of TIL is related to a better prognosis, especially in those tumours where there is a high CD8+ TIL/Treg ratio. Nevertheless, although in a high abundance, the mere presence of these cells does not always ensure a good outcome and vice versa. This highlights the need to deeply understand the cellular subtypes infiltrating the tumour and their activation status, specificity, and function. Since antigen-specificity of T cells is provided by the TCR, it represents a promising prognostic biomarker. New high-throughput sequencing (HTS) technologies allow the gathering of high quantities of information about the TCR repertoire of TIL, although there is still a lack of knowledge in this field. The most studied feature of the TCR repertoire is probably the diversity, as it reflects the number of different clonotypes and their abundance, i.e., diversity can give information about TILs undergoing clonal expansions, that indicate activation and response of T cells. Thus, the TCR repertoire is the reflection of processes that have shaped it. However, it is still unclear how the TCR repertoire should be configured in patients with cancer to indicate a better prognosis of the disease or better response to treatments. Our aim is to review the most important TCR repertoire studies in cancer to date to try to elucidate conclusions in this field.

## 2. T Cell Receptor

### 2.1. TCR Assembly and Structure

TCRs are transmembrane glycoprotein heterodimers formed by a combination of alpha and beta chains (TCRαβ), or gamma and delta chains (TCRγδ). They are exclusively expressed in T cells, and most of them (95%) consist in the TCRαβ heterodimer. The TCRα and TCRβ ectodomains are composed of a variable domain (Vα and Vβ), which is critical for peptide recognition, and a constant domain (Cα and Cβ).

**Figure 1:**
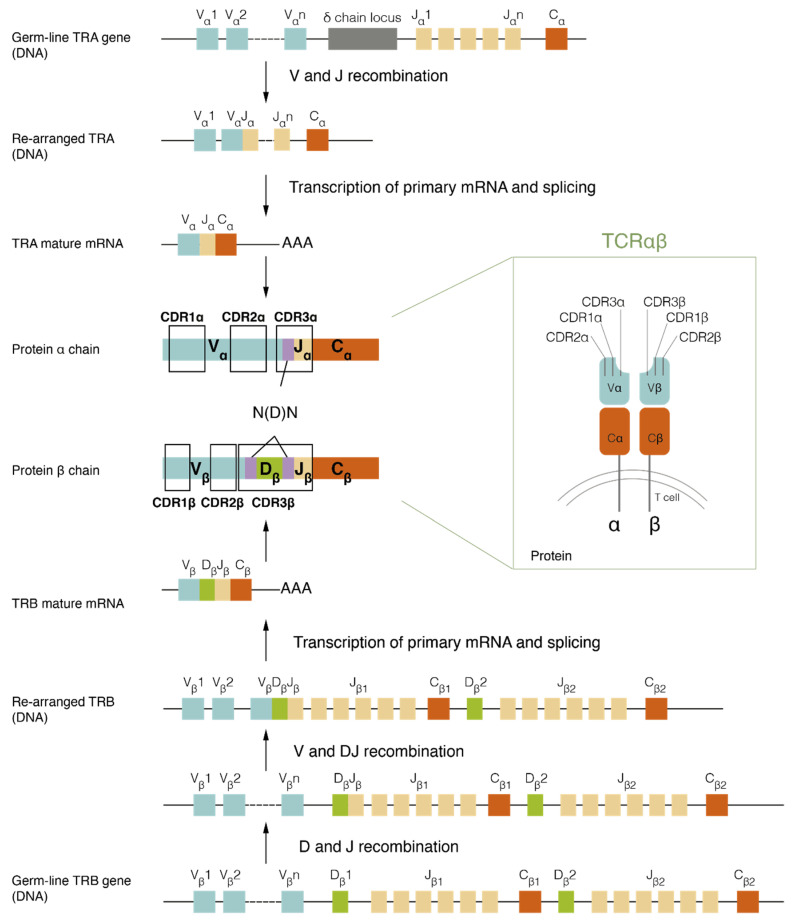
TCR structure and generation. The TCRαβ complex is composed of an alpha and a beta chain, each constituted by a variable (Vα and Vβ) and a constant domain (Cα and Cβ). The variable domain is generated through the variable (V), diversity (D) and joining (J) segments recombination. Vα and Jα segments are recombined in the alpha chain (TRA gene); Vβ, Dβ, and Jβ segments are recombined in the beta chain (TRB gene). Three complementary determining regions (CDRs) are contained in both variable domains: CDR1 and CDR2 are germline-encoded, and CDR3, which suffers random addition and deletion of nucleotides, is contained in the recombined V(D)J region. Image created by the authors.

Genes encoding for α and β chains (TRA and TRB, respectively) are composed of multiple non-contiguous segments: variable (V) and joining (J) segments for TRA and TRB, and diversity (D) segments for TRB. TCR genes undergo V(D)J recombination: random V and J segments for the alpha chain, and V, D, and J segments for the beta chain recombine among them to form the coding sequence for a functional variable domain. In turn, this sequence recombines with a C segment, and it is finally transcribed into a functional TRA or TRB chain transcript. Both TCRα and TCRβ chains contain three hypervariable loops in their structure, named complementary determining regions 1, 2, and 3 (CDR1, CDR2, CDR3) [1,2]. The CDR1 and CDR2 regions are germline-encoded by the V segments. The CDR3 region is encoded by the junctional regions V(D)J, where random addition, deletion, or both, of nucleotides (N) at the junction sites between segments are produced–(N(D)N) inserts [3,4]–providing a high hypervariability to this region [5,6]. These processes end up generating a TCR comprised of specific α and β chains that determine the specificity of the receptor (Figure 1).

The combinatorial variability of V(D)J recombination of TRA and TRB, the N(D)N generation, and the following heterodimeric pairing of different α and β chains generates a wide variety of different TCRs, ensuring the recognition of a vast variety of antigens. The overall population of unique TCR sequences is known as the TCR repertoire. It is shaped early in the thymus, where the thymic epithelial cells presenting peptides bound to the major histocompatibility complexes (MHC) expressed—named human leukocyte antigens (HLA) in humans—contribute to the development of T cells. Immature thymocytes–precursors of T lymphocytes—expressing TCRs that interact with peptide—MHC (pMHC), but do not strongly react against self-peptides, survive and emigrate to the periphery [7]. In contrast, thymocytes unable to recognise MHC or that strongly react against self-peptides will die via apoptosis. This process is part of the central tolerance to prevent that T cells react against self-proteins. Therefore, the peripheral TCR repertoire is composed of TCRs that recognise with low affinity the self-pMHC complexes. It is also during the thymic selection that lymphocytes are differentiated on CD4+ or CD8+ T cells, depending on the MHC molecule that they have recognised, class II (MHC-II) or class I (MHC-I), respectively.

The theoretical variability of the TCR is between 10^15^ and 10^20^. However, although about 3% of thymocytes are selected in the thymus and migrate to the periphery, the real diversity in humans is much lower, estimated at approximately 2 × 10^7^ different clonotypes [8]. Numerous studies have explained this phenomenon by demonstrating that recombination processes are not completely random, although the exact reasons are still unknown, i.e., the V-region usage during V(D)J recombination is partially biased, and genetic and epigenetic factors also influence the composition of the pre-selected TCR repertoire [9]. In addition, exposure to certain antigens throughout a lifetime implies the expansion of specific TCR clonotypes. Finally, other processes involving immune suppression, such as cell transplantation [10,11], can generate a loss of diversity. Overall, the TCR repertoire of individuals is dynamic during their lifetime, decreasing with age and being conditioned by the exposure to antigens [9].

### 2.2. TCR Sequencing and Analysis

Until the development of next-generation sequencing (NGS), the number of peripheral TCR clonotypes was theoretically estimated but advances in these technologies have allowed a more accurate characterisation. Latest conclusions regarding the TCR repertoire, as the bias toward certain V-J rearrangements or the greater overlap between individuals than previously thought, have been obtained as a result of deep sequencing technologies [12]. The increasing number of sequencing technologies and bioinformatic tools for the TCR repertoire study is generating a great variability between different data obtained. Proof of this variability, and that it should be considered, is the number of systematic comparisons and reviews published in the last years, both comparing methods [13,14,15,16,17] and bioinformatic tools [18,19]. Results can vary due to several factors, which could be briefly summarised in four categories: the starting material, both the amount and nucleic acid used (gDNA or RNA); the use of bulk populations versus the single-cell analysis; the library preparation approach, multiplex-PCR-based or 5′RACE; and the different bioinformatic tools, especially used to lately correct or minimise the PCR and sequencing errors.

Many of the differences reported in these reviews are closely related to the diversity measurement. First, the use of gDNA or RNA can lead to different diversity values. gDNA-based methods do not give information on the expression level, are less sensitive, do not consider allelic exclusion and may lead to errors due to residuals V(D)J rearrangements [9,15], which may result in an overrated diversity measure. The most remarkable difference between bulk-population analysis and single-cell analysis is that this latter allows the sequencing of both the alpha and the beta chain in a single cell, known as αβ pairing [13,14]. This affects the diversity measure since the same beta chain can be paired with different alpha chains in different cells, and vice versa [20]. Thus, considering the chains separately can underestimate the diversity. It should be noted that single-cell methods cover a more limited number of cells than bulk approaches, thus also influencing in the results. Regarding the library preparation methods, the main difference is that multiplex-PCR-based methods do not allow the amplification of new allelic variants since it uses primers of all the known V genes. In contrast, the 5′RACE technologies use the terminal transferase activity of the reverse transcriptase enzyme, allowing the amplification of unknown variants [21]. Thus, the multiplex-PCR methods can also underestimate the diversity, as certain clonotypes may not be amplified. Both technologies are subjected to the PCR-produced bias [22], although less number of cycles are necessary in the 5′RACE approaches, minimising this effect [14].

Over time, some of the original methods have been improved, for example, by using unique molecular identifiers (UMIs) that eliminate the PCR-produced bias [23]. UMIs are small sequences incorporated during the RNA retro transcription, thus tagging each mRNA with a unique sequence that will be later amplified with the transcripts during the PCR. This allows to identify whether certain sequences were originally present at high abundance—identical sequences with different UMIs—or the same sequence is highly abundant because it was preferentially amplified by the PCR—equal sequences with the same UMI. Recent advances in TCR repertoire analysis now allow to combine single-cell TCR sequencing with transcriptomics analysis [24,25,26,27]. This has been specially interesting to link signatures of tumour-reactive T cells with their TCRs, although usually the aim of using these technologies is not to analyse diversity but to describe tumour-specific TCRs [28]. Even further improved, spatial transcriptomics, developed by Stahl PL et al. [29], now allows to perform a transcriptomic analysis in situ, with the aim to retain the cellular location information. There are still few cancer studies in which the TCR analysis and spatial transcriptomics are combined [26,30,31,32], but it will likely be increasingly used in the future.

Last, but not least, different classical variables defining populations are used for the analysis of TCR repertoires: richness, the number of different clonotypes in a sample; evenness, the relative abundance of these clonotypes; and diversity, the most used measurement, that combines richness and evenness, thus considering the number of clonotypes and how evenly they are distributed in a sample. A high diversity represents a high number of different clones in similar frequencies. On the contrary, a low diversity–high clonality expresses a reduced number of different clonotypes with much higher frequencies. Diversity and clonality can be measured by several indices, although they are usually measured by the Shannon’s diversity index (also known as Shannon’s entropy and Shannon–Wiener index), the Simpson index, the inverse Simpson index, and the Gini–Simpson index. Some authors also include other metrics, such as: the High-Expanded Clones (HEC) ratio, calculated as the absolute number of clones with a frequency over a certain percentage (usually 0.5%); and the U/T index, calculated as the number of productive, unique sequences, divided by the total number of sequences. These and other statistical indices have also been reviewed before [15,16,18], but, again, as several indices can be used, a great variability is generated in the results obtained by different studies. It has been recently proven by Chiffelle et al. [15] that different results are retrieved by applying different metrics in a particular sample.

Although most of the existing technologies, statistical approaches, and bioinformatics tools are valid and approved, the main problem lies in the great variability generated by combining all these factors. Altogether, it becomes difficult to compare the data published in the literature, in this case arising from cancer studies. Standardisation of the TCR repertoire analysis would be valuable to skew the variability.

## 3. T Cell Response to Different Type of Tumoural Antigens

In general, during the immune response, specific T cells that recognise antigens presented by antigen-presenting cells (APC) are activated in the lymph nodes (LN), undergo clonal expansion, and migrate to the injured tissue. Antigens presented by HLA-II molecules activate CD4+ T cells, that differentiate into T helper cell subsets, while antigens presented by HLA-I molecules stimulate the differentiation of CD8+ T cells into cytotoxic T cells. T cells must encounter the same antigens in the tissue to identify infected or injured cells and execute their effector functions. As all the clonotypes from a clonal expansion carry an identical CDR3, this can be used as an identifier to track specific cells.

However, finding specific clonotypes in tumours can be more complex than in other contexts such as infections. On the one hand, most of the antigens resulting from the destruction or apoptosis of tumour cells will be self-peptides, so T cells will theoretically remain tolerant against them. Nevertheless, certain differences between tumoural and normal tissues, given the intrinsic nature of tumoural cells, could favour T cell recognition. On the other hand, activated clonotypes that migrate to the tumour site must encounter tumoural cells presenting the antigens to effectuate an anti-tumoural response. As epithelial cells only express HLA-I molecules, they can only be directly recognised by CD8+, but not CD4+ T cells. Thus, the maintenance of CD4+ T cells depends on the presence of APC, i.e., B cells, macrophages, and dendritic cells (DC), in the tumour site. Although generally CD4+ T cells do not have a cytotoxic function, their role in cancer has been recently reconsidered, as they are necessary for a proper activation of the immune response and the maintenance of the immunological memory [33]. Beyond this, tumoural epithelial cells can downregulate HLA expression, so APC become even more necessary also to maintain CD8+ T cells.

As T cell responses depend on the antigen recognition, the type of tumour antigens presented by cancer cells may shape the TCR repertoires observed. Tumour antigens are usually divided in self-peptides and non-self-peptides, also named tumour-associated (TAAs) and tumour-specific (TSAs) antigens, respectively.

### 3.1. Tumour-Associated Antigens Recognition

TAAs have been widely studied as candidates for immunotherapy approaches. These are derived from self-proteins and can be categorised into cancer germline antigens, differentiation antigens, and overexpressed antigens [34,35].

Cancer germline antigens derive from proteins that are naturally expressed during foetal development and in certain types of tumours but are usually unexpressed in adult normal tissues. The most investigated cancer germline antigens are NY-ESO-1 and MAGE-A antigens, first reported in patients with synovial cell sarcoma or melanoma [36,37,38,39,40,41], but also described in several other types of tumours [38,42,43,44,45,46,47,48,49,50,51,52,53]. Differentiation antigens are specific proteins from tissue or cells, both healthy and affected, where the tumour is occurring, but that are not expressed in other tissues, as CD19 in most of B cell lymphomas [35,54] or gp100 and MART-1 in melanoma [55,56]). Overexpressed antigens are proteins found in several tissues which are highly expressed in the tumour, as ERBB2 in breast and ovarian cancers [57,58]).

The great advantage of using TAAs as targets is that they are shared between individuals, so individualised treatments are not required. However, targeting self-proteins implies toxicities due to the cellular death of healthy cells too, which may vary from a temporal loss of the target cells to permanent destruction of certain tissues [35,59]. Finally, although the binding affinity of TCR to the self-peptides-MHC complexes has been reported to be much lower than to foreign antigens [60], the release of self-antigens in high concentrations may activate a high-dose/low-affinity response. This could compromise the peripheral tolerance, triggering autoimmune events [61].

### 3.2. Tumour-Specific Antigens Recognition

In the 1950s, Prehn et al. [62] proposed that non-synonymous mutations on the DNA could produce mutated proteins expressed by tumours. Later, it was hypothesised that these proteins could generate different peptides able to activate specific T cells. These mutated self-peptides are known as neoantigens, and due to the advances in sequencing technologies, they are probably the most popular candidates for targeting tumoural cells. Neoantigens are then TSAs, as they are only expressed in tumoural tissues. TSAs also include viral antigens, which are expressed and presented in cancer cells with an oncovirus origin [63]. In contrast to TAAs, neoantigens are individual and specific since mutations randomly occur, thus limiting the widespread use of treatment. Targeting TSAs is supposed to be safer, as these antigens should not be presented by normal cells and tissues [35].

The HLA haplotype of the individual restricts antigen presentation, and each HLA can only present peptides with a certain motif. A mutation in the DNA sequence does not directly imply a presentation of the corresponding aminoacidic sequence, and at the same time, not all the mutations imply peptides with higher HLA affinity. The loss of distinct amino acids in some positions could produce the opposite effect as certain HLA pockets are highly restricted to specific amino acids [64,65]. In addition, a high abundance of neoantigens would be required to occupy a relevant part of the presented peptidome. Moreover, their expression should be maintained over time to orchestrate a good immune response. Therefore, the number of neoantigens presented by HLA molecules would probably be less favoured than that of TAAs, as these latter are highly or constitutionally expressed in tumours. This implies that finding neoantigens by whole-exome sequencing should be accompanied by studies on their expression over time and whether they are processed and presented by APC, considering the HLA restriction of the individual.

## 4. The TCR Repertoire as a Prognostic Biomarker in Cancer

In sum, the existence of certain peptides that could trigger an anti-tumoural response would imply T cell expansion. However, such expansion can be limited by various factors: (i) the presence of APC; (ii) the tumoural antigen exposition over time; (iii) the HLA restriction of the individuals, that at the same time implies certain clonal restriction; and (iv) the TCR repertoire status of the patients at the time of the disease. All these factors condition the TCR diversity, which can be monitored by TCR HTS, a potent tool to improve the understanding of the T cell responses in many contexts [66]. Several TCR repertoire studies investigating anti-tumoural responses are summarised below (Table 1).

### 4.1. TCR Repertoire in Patients with Cancer

Cancer studies using peripheral blood have reported that high diversity in the TCR repertoire may be associated with better prognosis. For example, in stage I–IV melanoma patients, high diversity has been directly associated with longer progression-free survival (PFS), although without any impact on overall survival (OS) [67]. Furthermore, in a subgroup of patients with breast cancer with combined lymphopenia (low number of lymphocytes) and divpenia (low TCR diversity), a particularly elevated risk of early death was reported [68]. More specifically, in cervical cancer, it has been demonstrated that the peripheral diversity gradually diminishes as the carcinogenesis progresses: patients with cervical cancer presented the least diverse repertoire, followed by patients with cervical intraepithelial neoplasia, and finally healthy donors [69]. However, other studies have reached opposing conclusions: patients with nasopharyngeal carcinoma presented a lower percentage of HECs in the peripheral blood, thus a higher diversity, than healthy individuals or disease-control individuals (patients with other nasopharyngeal disease) [70]. Finally, in a pancreatic cancer study, no differences in the peripheral diversity were observed between patients and healthy individuals [71]. To conclude, the use of peripheral blood samples to study TCR repertoire may be attractive, given its ease of obtaining; however, intra-tumoural and peripheral TCR repertoires are different [71,72,73,74] and this can cause biases. In particular, in peripheral repertoires, the abundance of tumour-specific lymphocytes may be diluted, and the TCR diversity may be affected, as previously stated, by other factors such as age, previous exposure to different antigens, or underlying immunosuppression processes.

In this context, the analysis of TCR repertoire at the tumour site may provide more detailed information about the immune response, although there are controversial data from multiple studies comparing the TCR repertoires of the tumour and of the adjacent normal tissues. Some studies have shown more diverse TCR repertoires in the tumour site in comparison to the normal tissue (breast [75] and hepatocellular cancers [76]); others have found no differences (hepatocellular cancer [77], gastric cancer [78], oesophageal squamous cell carcinoma [79], and nasopharyngeal carcinoma [70]); and others have reported less diverse repertoires in tumours (breast [80], gastric [81], and colorectal cancers [82]). However, the studies that have associated intra-tumoural TCR diversity with outcome have reached the same conclusion: lower diversity levels are associated with a worse prognosis [70,81,83]. In addition, not only the diversity but also the level of similarity in TCR sequences between the tumoural and the normal tissues have been demonstrated to give information about the patients’ outcomes. Some authors have reported that a greater overlap (i.e., a higher number of shared TCR sequences) is related to a better outcome. Moreover, similarities have been observed to gradually decrease during tumourigenesis [78]. Thus, a decrease in the number of different clones, causing a decrease in TCR diversity, also provokes the normal and tumoural TCR repertoires to become more different when the disease worsens. This may indicate that changes in the malignant cells elicit an adaptation in the T cell response and, consequently, a modification in the TCR repertoire.

Overall, it seems that there is a higher TCR diversity in healthy individuals or patients with better disease progression. Indeed, a higher TCR diversity indicates a functional immune system with a better capacity to orchestrate an anti-tumoural response. At the same time, loss of diversity may be a consequence of an aggressive tumour, leading to a failure of the immune system.

It should be considered that data arising from different types of tumours is not always comparable, as differences between tissues may affect the TCR diversity. Evidencing this, it has been described that tissue-resident memory T cells, which are naturally present in tissues in healthy conditions, present a more clonal repertoire [84,85]. It can be deduced that the amount of this cellular subset may be affecting the global TCR repertoire diversity. Moreover, in a study performed by Keane et al. [83], different diversity levels were observed when comparing different types of tumours (i.e., patients with melanoma presented higher clonality than B cell lymphoma patients). Thus, other factors may be particularly relevant and explain the opposed data in different works.

**Table 1:** TCR repertoire results in different cancer studies.

Disease	Compartment	TCR Repertoire Results	Prognosis Association	References
Melanoma	PBMC	No differences between age or clinical stage and diversity.	High diversity associated with longer PFS.	Charles et al. [67]
	Metastatic LN	nr	High diversity of metastatic LN/PBMC ratio associated with better prognosis.	
Breast Cancer	PBMC	Inverse correlation between TCR diversity and age.	Low diversity combined with lymphopenia in patients with elevated risk of early death	Manuel et al. [68]
Ovarian Carcinoma	PBMC	PBMC showing TCR repertoires quite distinct from the tumour tissue.	nr	Emerson et al. [72]
	Tumoural tissue			
Cervical Cancer	PBMC	No differences between age and TCR diversity.	Diversity in PBMC decreasing as the carcinogenesis progressed. Lower diversity in the PBMC of CC, followed by CIN and healthy donors.	Cui et al. [69]
	Sentinel LN	nr	Lower number of clones in the sentinel LN indicating a worse prognosis.	
Breast Cancer	Tumoural and healthy tissue	Higher T cell infiltrates and TCR diversity in tumour than in healthy tissue.	nr	Wang et al. [75]
	LN	Higher diversity in the LN than in tumours or healthy tissue.		
NPC	PBMC	Higher diversity in NPC patients than healthy individuals.	Higher diversity in PBMC related with worse prognosis.	Jin et al. [70]
	Tumoural and healthy tissue	No differences between healthy and tumoural tissue.	Lower tumour/healthy diversity ratio associated with worse prognosis.	
HBV-associated HCC	Tumoural and healthy tissue	Higher diversity in tumoural tissue than in healthy tissue.	nr	Chen et al. [76]
Breast Cancer	Tumoural tissue	Lower diversity in tumoural tissue than in normal tissue.	nr	Beausang et al. [80]
Gastric Cancer	Tumoural tissue	Higher diversity in the adjacent mucosa than in tumoural tissue.	Diversity in the tumour not having an impact on the survival rate.	Jia et al. [81]
	Adjacent mucosa		Low diversity in the adjacent mucosa related with a poor clinical prognosis.	
	PBMC	Higher diversity in PBMC than in tumoural tissue.	Diversity in PBMC not having an impact in the survival rate.	
Diffuse Large B-Cell Lymphoma	DLBCL nodes and non-diseased nodes	Lower diversity in DLBCL nodes than in non-diseased nodes.	Lower diversity is associated with adverse outcomes.	Keane et al. [83]
Colorectal Cancer	Tumoural tissue and adjacent mucosa	Higher diversity in the adjacent mucosa than in tumoural tissue.	nr	Sherwood et al. [82]
HBV-associated HCC	Tumoural and adjacent tissue	No differences between tumoural and adjacent tissue.	Diversity not correlated with the progression of the disease.	Lin et al. [77]
			Lower overlap between healthy and tumoural tissue observed in patients with shorter PFS.	
Gastric Cancer	Tumoural tissue and adjacent mucosa	No differences between tumoural and adjacent tissue.	Tumoural and adjacent mucosa overlap gradually decreasing during gastric carcinogenesis.	Kuang et al. [78]
OSCC	Tumoural and adjacent tissue	No differences between tumoural and adjacent tissue.	nr	Chen et al. [79]

CC, cervical cancer; CIN, cervical intraepithelial neoplasia; HBV, hepatitis B virus; HCC, hepatocellular carcinoma; LN, lymph node; nr, not reported; NPC, nasopharyngeal carcinoma; OSCC, oesophageal squamous cell carcinoma; PBMC, peripheral blood mononuclear cells; PFS, progression-free survival; TCR, T cell receptor.

### 4.2. Determinant Factors of the TCR Repertoire

The wide variability of results obtained from different studies may be explained by several factors not yet extensively studied. There is a common tendency to focus on certain study areas, such as the levels of TIL, the Treg+/CD8+ ratio, or the TCR repertoire diversity, as independent events, but the immune response should be studied as a whole. In the specific case of the TCR repertoire in cancer, two major factors can explain variabilities: the level of immune tumoural infiltration and the tumoural mutation burden (TMB). It has been previously proposed that tumours can be classified according to these two parameters [86], and some studies are now including them among the factors to identify statistically significant differences to predict cancer prognosis and progression [87,88,89,90].

On the one hand, intrinsic differences between tissues condition the repertoire, as different levels of infiltration are naturally present. Recently, Marderstein et al. performed an exhaustive study on the immune cells’ infiltration comparing different tissues and individuals [91]. First, cancer tissues can be divided into hot and cold by their infiltrating numbers but also by their cellular composition. Marderstein’s group performed hierarchical clustering of tissues based on the quantity of 14 different immune cell types from innate and adaptive immunity. They observed that certain tissues across the body cluster closely, such as skin, gastroesophageal, or brain sub-tissues. At the same time, other tissues part of the same system, cluster distinctly, i.e., transverse colon, sigmoid colon, and small intestine tissues presented very different immune infiltrates, especially in the T cell content. Moreover, certain tissues, such as lungs are highly infiltrated by macrophages, which in the tumour microenvironment are known to be a double-edged sword: they can phagocyte cancer cells or act as T cell recruiters, but also can induce angiogenesis and promote tumour invasion [92,93]. All these intrinsic differences should be worth considering in cancer, as different basal infiltrate levels of different immune populations will certainly orchestrate different anti-tumoural responses. Second, different individuals can have very different infiltration levels, so the disparity of conclusions in the TCR repertoire studies may also be due to these variances.

On the other hand, the TMB may be a key conditioner of the TCR repertoire and may explain why different clonality levels are observed in tumours in comparison to the unaffected tissues in some studies. Many mutations increase the probability of genes being translated in neoantigens with higher HLA affinity. This will influence the number of clonal expansions, as the chances of T cells recognising some peptides will also be increased. The loss of TCR diversity has been associated several times with a worse prognosis. Moreover, tumours with a high TMB are more aggressive, and are associated with more clonal expansions. As higher clonality does not translate into an improvement of the patients’ condition, this raises the question of whether expanded clonotypes recognise target cells. It has been demonstrated in patients with melanoma that clones present in higher frequencies can recognise neoantigens and TAAs [94]. Indeed, expanded clones in tumours exhibiting a high TMB, such as melanoma, may be recognising tumour-derived peptides, but they might be inhibited by programmed cell death protein 1 (PD-1), or cytotoxic T-lymphocyte-associated protein 4 (CTLA-4). In line with these findings, several studies have demonstrated that patients with higher clonality are the best responders to anti-PD-1 treatment [95,96]. Moreover, tumours with high TMB due to mismatch-repair deficiency, strongly associated with microsatellite instability such as certain colorectal tumours, have been reported several times to be associated with high immune infiltration and better ICI responses [97,98,99,100,101,102,103,104,105,106,107]. This reinforces the idea that a high TMB generates more neoantigens, which increase the infiltration and activation of tumour-specific TILs.

## 5. The TCR Repertoire as a Predictive Biomarker of Immune Checkpoint Inhibitor Treatments

Over the last 10 years, the use of ICI has revolutionised the field of cancer treatment. The authorisation of anti-CTLA-4 (ipilimumab) as the first antibody blocking an immune checkpoint was shortly followed by the use of blocking antibodies for PD-1 (pembrolizumab and nivolumab) and programmed death-ligand 1 (PD-L1) (atezolizumab, durvalumab and avelumab). This has been followed by an impressive number of clinical trials being carried out [108]. CTLA-4 is expressed in T cells and competes with the co-stimulatory receptor CD28 for its ligands CD80 and CD86 [109]. CTLA-4 has a higher affinity for these ligands, but unlike CD28, it is not constitutively expressed in T cells, except for Treg [110]. Its expression is quickly upregulated after the engagement of TCR by cognate pMHC, serving as a co-inhibitory signal for the termination of T cell activation. CTLA-4 knockout mice develop a systemic immune hyperactivation [111], demonstrating its critical role in restraining the immune system. Differently, the PD-1 interacts with PD-L1 or PD-L2 to avoid exacerbated responses produced by persistent exposure to antigens [112]. As mentioned, chronic antigen exposure occurs in cancers and can break the peripheral tolerance. Thus, the expression of PD-1 in previously activated T cells is an immune checkpoint to control self-reactive responses, leading to an “exhausted” phenotype.

ICI are used to treat several cancers, such as melanoma; non-small cell lung cancer; urothelial cancer; squamous cell carcinoma of head and neck; renal cell, gastric, hepatocellular, breast, and Merkel-cell carcinomas; and Hodgkin lymphoma [113]. Despite this fact, the success of ICI is not widespread among patients and even less so among different types of cancer. Indeed, the use of ICI or other immune-related treatments implies a direct alteration in the immune system, and new treatments cause undesired events, now known as immune-related adverse events (irAEs). A side effect of the activation of the immune system, irAEs are autoimmune-like responses that can occur in any organ or system [114,115,116]. The ICI response and irAEs are immunologically driven, with T cells having a critical role. By extension, the TCR has turned into one of the best candidates to be a predictive biomarker for ICI response, as evidenced by its use in several studies summarised in this review (Table 2).

The use of anti-CTLA-4 is thought to increase the peripheral TCR diversity [118,122,127,132], whereas this effect is not as clear when using anti-PD-(L)-1 (refers to both PD-1 and PD-L1) [120,123]. In addition, a higher TCR diversity at baseline has been associated with improved survival after anti-CTLA-4 treatment, indicating that TCR could be used as a predictor of potential responders [117,118,119,120,121,124,125,136]. Regarding anti-PD-(L)-1 therapies, some authors have also described an association of higher peripheral diversities at baseline with better outcomes, both using anti-PD-1 [117,128,129] and anti-PD-L1 [131], but the opposite associations have also been reported [119]. It has also been suggested that the efficacy of ICI therapies may be associated with age, as elderly people present a reduced thymic functionality and a lower peripheral TCR diversity [137]. This would be in concordance with the idea that more diversified TCR repertoires increase the likelihood of tumour antigens recognition. However, clinical data from other studies reveal that there are no differences between young and old patients responding to ICI therapies [138,139,140].

Post-treatment studies have been performed distinguishing long-term survivors (or responders) from short-term survivors (or non-responders) to understand the modulation of the TCR repertoire in both subsets. Whereas the use of anti-CTLA-4 has been reported to increase the TCR diversity [118,122,127,132], some authors have described that among patients receiving this ICI treatment, responders were those presenting less TCR diversification [120,126]. However, other authors have described diversity levels to be maintained in patients with an improved clinical outcome [118]. Again, the disparity of results obtained in different studies manifest the need to better understand the changes in the TCR repertoire which may be affected by other factors, as previously stated.

Higher TCR clonality in the peripheral or the intra-tumoural TCR repertoire after anti-PD-(L)-1 immunotherapy has been observed in long-term survivors or associated with an increased PFS in pancreatic ductal adenocarcinoma [120], melanoma [133], non-small cell lung cancer [128], and urothelial carcinoma [131]. The inverse effect (higher diversities in the responder group or patients with longer OS) has been reported in other studies in the peripheral repertoire of non-small cell lung cancer [130], and in the intra-tumoural repertoire of Merkel cell carcinoma [135].

Differences in the mechanisms of CTLA-4 and PD-1, although both have an inhibitory effect on T cells, are essential to understand the consequences on the TCR repertoire, especially in the periphery. Anti-CTLA-4 targets almost all T cells, allowing activation and proliferation of several clonotypes in the LN, and finally broadening the peripheral TCR repertoire. In contrast, the blocking antibodies for PD-(L)-1 specifically act in previously primed cells, and a reshaping of the peripheral TCR repertoire is less likely to occur, and it will have a localised effect. Several studies have suggested that pre-existing T cell responses correlate with better outcomes after anti-PD-1 therapies [95,117,141]. Furthermore, toxicities after the use of anti-CTLA-4 are more usual and severe when compared with anti-PD-1/PD-L1 immunotherapy [142]. Finally, toxicities are even more common (nearly 60%) in patients treated with combined therapies (anti-PD-1 plus anti-CTLA-4) [143,144].

ICI response, irAEs development, and changes in TCR repertoire are all interconnected events. Anti-CTLA-4-induced irAEs have been associated with both favourable outcomes [113] and greater TCR diversifications [122,127]. This suggests that the increase in the TCR diversity concurrently facilitates the tumour-reactive and the self-reactive T cell activation and expansion. As anti-PD-(L)-1 affects the primed T cells, the use of the TCR as a biomarker needs to be combined with other factors, as combination of multiple parameters has been reported as an accurate predictor of irAEs across multiple tumour types [145].

## 6. Conclusions

Although there is still a lack of knowledge about the TCR repertoire during cancer evolution and whether or how other factors may influence it, there is clear evidence that the TCR repertoire has great potential as a biomarker. Until now, TIL quantity has been used to determine patients’ outcomes; however, high-quality immune responses are also necessary. This review focused on the use of the TCR diversity as a quality measurement, as determining the presence/absence of clonal expansions is necessary to understand the specificity of the response. The study of specific tumoural sequences, especially those shared among individuals, also known as public TCR, could improve TCR-based therapies. In summary, most of the studies suggest that a high diversity may be a good indicator of a quality response: it not only suggests a healthy and functional immune system, but also clonotype diversification increases the possibilities to encounter and recognise tumoural antigens. Moreover, high diversity has also been reported as a good predictive biomarker for anti-CTLA-4 response, demonstrating that diversified instead of restricted responses are mainly more efficient. However, under certain circumstances, such as tumours with a high TMB, high clonality may be indicating a neoantigen response. In the near future, immune monitoring should not only consider tumour microenvironment and TMB, but also immunologic infiltration, immune phenotype, and TCR diversity, to decide the best treatments in different patients and tumours and beneficially modulate the immune response.

## Figures and Tables

**Table 2:** TCR repertoire analysis after ICI treatment in different cancer studies.

				Association with ICI Response			
Disease	Compartment	ICI	Effect of ICI	TCR Repertoire at Baseline	TCR Repertoire after Treatment	Development of irAEs	References
Melanoma	PBMC	a-CTLA-4	nr	High diversity at baseline in LTS.	nr	Highly restricted TCR repertoire in patients developing irAEs.	Arakawa et al. [117]
		a-PD-1	nr	High diversity at baseline in LTS.	nr	nr	
Melanoma	PBMC	a-CTLA-4	Increase in diversity.	High diversity at baseline associated with improved survival.	Reduced clonotype loss associated with improved clinical outcome.	nr	Cha et al. [118]
Prostate Cancer	PBMC	a-CTLA-4	Increase in diversity.	High diversity at baseline associated with improved survival.	Reduced clonotype loss associated with improved clinical outcome.	nr	
Melanoma	PBMC	a-CTLA-4	nr	High diversity at baseline associated with improved survival.	nr	nr	Hogan et al. [119]
		a-PD-1	nr	Higher clonality at baseline associated with major pathological response.	nr	nr	
Pancreatic Ductal Adenocarcinoma	PBMC	a-CTLA-4	No significant changes.	High diversity at baseline associated with improved survival.	LTS showing a higher number of expanded clones after treatment.	nr	Hopkins et al. [120]
		a-PD-1	No significant changes.	nr	LTS showing higher clonality after treatment.	nr	
Melanoma	PBMC	a-CTLA-4	nr	High diversity at baseline associated with improved survival.	nr	nr	Postow et al. [121]
Melanoma	PBMC	a-CTLA-4	Increase in diversity.	nr	Responders exhibiting an increase in TCR richness.	Higher diversity associated with increased toxicities.	Robert et al. [122]
Melanoma	PBMC	a-PD-1	No significant changes.	nr	Responders exhibiting both increase and decrease in richness indifferently.	nr	Robert et al. [123]
Prostate Cancer	PBMC	a-CTLA-4	nr	nr	nr	High CD8+ clonality related with irAEs.	Subudhi et al. [124]
Melanoma	PBMC	a-CTLA-4	nr	No association between pre-treatment and response.	Patients with higher diversity having longer PFS and OS.	nr	Khunger et al. [125]
	Tumoural tissue	a-CTLA-4	nr	Higher clonality at baseline associated with longer PFS and OS.	nr	nr	
Clear Cell Adenocarcinoma, Melanoma and Prostate Cancer	PBMC	a-CTLA-4	nr	nr	A trend toward higher clonality in responders.	nr	Looney et al. [126]
Prostate Cancer	PBMC	a-CTLA-4	Increase in diversity.	nr	nr	Higher diversity post-ICI/baseline ratio associated with irAEs.	Oh et al. [127]
NSCLC	PBMC	a-PD-1	nr	High diversity of PD-1+CD8+ at baseline associated with improved survival.	High clonality after treatment associated with increased PFS.	nr	Han et al. [128]
NSCLC	PBMC	a-PD-1	nr	nr	Responders exhibiting a higher expansion of peripheral clones previously found in the tumour.	nr	Forde et al. [129]
	Tumoural tissue	a-PD-1	nr	Higher clonality at baseline associated with major pathological response.	nr	nr	
NSCLC	PBMC	a-PD-L1	nr	nr	High diversity after treatment associated with longer OS.	nr	Naidus et al. [130]
Urothelial Carcinoma	PBMC	a-PD-L1	nr	High diversity at baseline associated with improved survival.	High clonality after treatment associated with increased PFS.	nr	Snyder et al. [131]
Breast Cancer	Tumoural tissue	a-CTLA-4	a-CTLA-4 alone expands intra-tumoural lymphocytes, increasing clonality. Cryoablation inducing polyclonality, independently from a-CTLA-4.	nr	nr	nr	Page et al. [132]
Melanoma	Tumoural tissue	a-CTLA-4	nr	Not significant results.	nr	nr	Roh et al. [96]
		a-PD-1 + a-CTLA-4	nr	Responders exhibiting a higher clonality at pre-a-CTLA-4 treatment.	nr	nr	
Melanoma	Tumoural tissue	a-PD-1	nr	nr	Responders exhibiting more oligoclonal expansions.	nr	Inoue et al. [133]
Glioblastoma	Tumoural tissue	a-PD-1	Increase in diversity.	nr	nr	nr	Schalper et al. [134].
Melanoma	Tumoural tissue	a-PD-1	nr	High clonality at baseline associated with improved survival.	Responders exhibiting more oligoclonal expansions.		Tumeh et al. [95]
Merkel Cell Carcinoma	Tumoural tissue	a-PD1 or	nr	nr	Responders exhibiting higher diversity.	nr	Spassova et al. [135]
		a-PD-L1					
Melanoma	Tumoural tissue	a-PD-1+	nr	Responders exhibiting a higher clonality at baseline.	Higher clonality correlating with clinical benefit.	nr	Yusko et al. [136]
		a-CTLA-4					

CTLA-4, cytotoxic T-lymphocyte-associated protein 4; ICI, immune checkpoint inhibitors; irAEs, immune-related adverse events; LTS, long-term survivors; nr, not reported; NSCLC; non-small cell lung cancer; OS, overall survival; PBMC, peripheral blood mononuclear cells; PD-1, programmed cell death protein; PFS, progression-free survival; TCR, T cell receptor.
